# Cervical length dynamics in triplet pregnancies: a retrospective cohort study

**DOI:** 10.1007/s00404-017-4402-0

**Published:** 2017-05-24

**Authors:** Sophie Pils, Stephanie Springer, Verena Wehrmann, Kinga Chalubinski, Johannes Ott

**Affiliations:** 1 Clinical Division of Gynecologic Endocrinology and Reproductive Medicine, Department of Obstetrics and Gynecology, Medical University of Vienna, Waehringer Guertel 18-20, 1090 Vienna, Austria; 2 Clinical Division of Obstetrics and Fetomaternal Medicine, Department of Obstetrics and Gynecology, Medical University of Vienna, Waehringer Guertel 18-20, 1090 Vienna, Austria

**Keywords:** Cervical insufficiency, Cervical length, Preterm delivery, Multiple pregnancy, Triplet pregnancy

## Abstract

**Purpose:**

To review our experience with a screening program that included sequential cervical length measurements in our large population of triplet pregnancies.

**Methods:**

Seventy-eight triplet pregnancies were retrospectively included. Cervical length measurements were performed by transvaginal ultrasound in 2-week intervals from week 16 + 0 onwards in a tertiary-care center in Vienna. The main outcome measurement was preterm delivery prior to 32 + 0 weeks of gestation. Statistical analyses were performed using paired and unpaired *t* tests and a stepwise linear regression model.

**Results:**

There were 26 cases of preterm delivery (33.3%). Women with preterm delivery revealed significant cervical length shortening from week 22 + 0 (median 33 mm, interquartile range, IQR 17–39) to 24 + 0 (median 21 mm, IQR 7–30; *p* = 0.005). This was not observed in women without preterm delivery. From week 22 + 0 onwards, both groups showed further significant 2-week differences in cervical length (*p* < 0.05). Univariate analysis of cervical length in weeks 20 + 0, 22 + 0, and 24 + 0 as well as cervical length dynamics from 22 + 0 to 24 + 0 predicted preterm delivery.

**Conclusions:**

In triplet pregnancies, a decrease in cervical length seems physiological from week 22 + 0 onwards. A sharp decrease in cervical length from the 22 + 0 to the 24 + 0 week as well as the smaller cervical length in weeks 20 + 0, 22 + 0, and 24 + 0 increase the risk of preterm delivery.

## Introduction

Over the past decades, the incidence of multifetal gestations has increased dramatically which also includes triplet pregnancies [1]. In these pregnancies, the average pregnancy age at delivery ranges from 31 to 33 completed weeks of gestation, with a majority of triplets being born before the 35th week [2–5]. It is evident that this prematurity is accompanied by high rates of neonatal complications which include both neonatal mortality and morbidity [6]. Although gestational hypertension and its complications, first and foremost preeclampsia, also contribute to the risk of preterm delivery in triplet pregnancies, the majority of cases is complicated by preterm labor and cervical shortening [5]. Notably, chorionicity has also been proven to be a risk factor for earlier delivery in triplets with dichorionic pregnancies having been reported to deliver more likely <30 weeks of gestation than trichorionic pregnancies [7]. Moreover, one could also hypothesize that chorionicity and/or the amount of amniotic fluid might influence cervical length (CL) in triplet pregnancies, probably even without having a major impact on gestational length. However, the efforts of identifying those women at risk for preterm triplet delivery have also focused on CL measurement [2, 8–16]. Despite the fact that these studies measured the CL at different pregnancy stages and resulted in different cut-off levels, they demonstrated that the CL can be used as a more or less reliable predictive tool. At the Medical University of Vienna, serial CL measurements of women at risk of preterm delivery (PTD) have been part of the clinical routine for a long time [17, 18]. This also applies to women with triplet pregnancies. We thus intended to critically review our experience with this diagnostic regimen. We aimed to evaluate the kinetics of CL and to test the predictive value of the CL at different gestational ages for the prediction of preterm triplet delivery before 32 + 0 weeks of gestation. In addition, we also focused on the influence of chorionicity on CL dynamics.

## Materials and methods

**Fig. 1:**
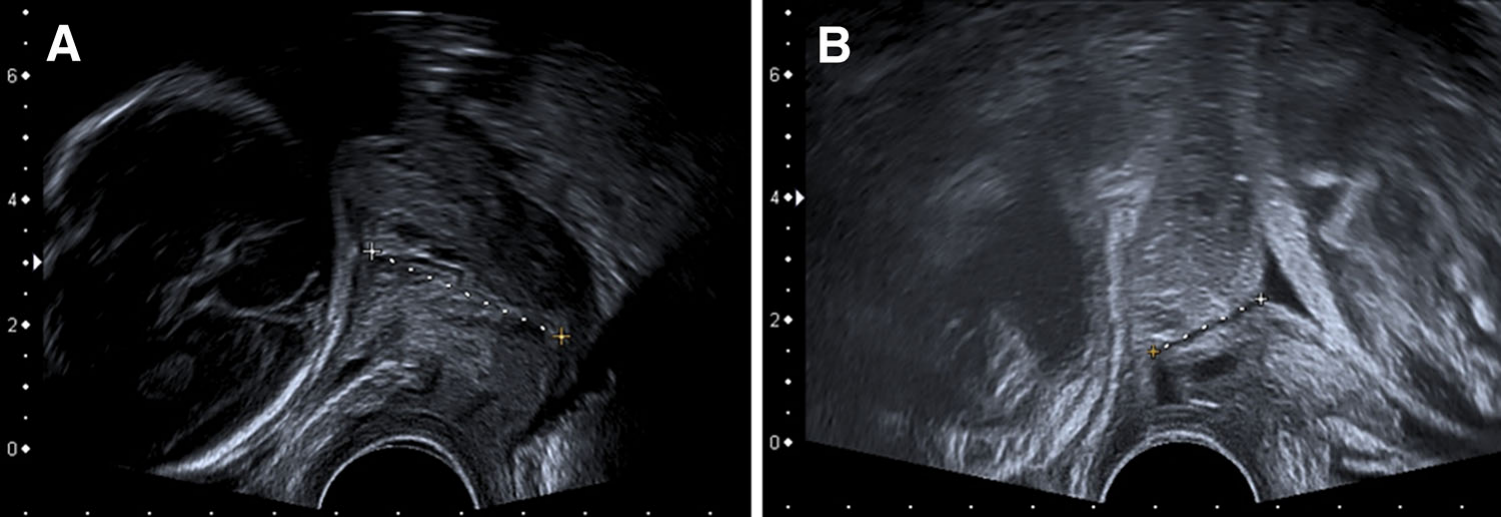
Sonographic cervical length measurement

As reported previously [17, 18], a screening program for pregnant women at perceived risk of PTD has been established for many years at the Department of Maternal-Fetal Medicine of the Medical University of Vienna, Austria. The department is the national reference center for maternal-fetal medicine in eastern Austria and the annual number of deliveries was at least 2500 during the study period. The screening program included CL measurement by transvaginal ultrasound every 2 weeks from 16 + 0 until 30 + 0. All ultrasound examinations were performed by highly experienced operators (either obstetricians or certified medical technical assistants). All CL measurements (Fig. 1) were carried out according to the guidelines of the Fetal Medicine Foundation (available online at http://www.fetalmedicine.com/fmf/training-certification/certificates-of-competence/cervical-assessment/). The shortest of at least three measurements was documented [18].

**Fig. 2:**
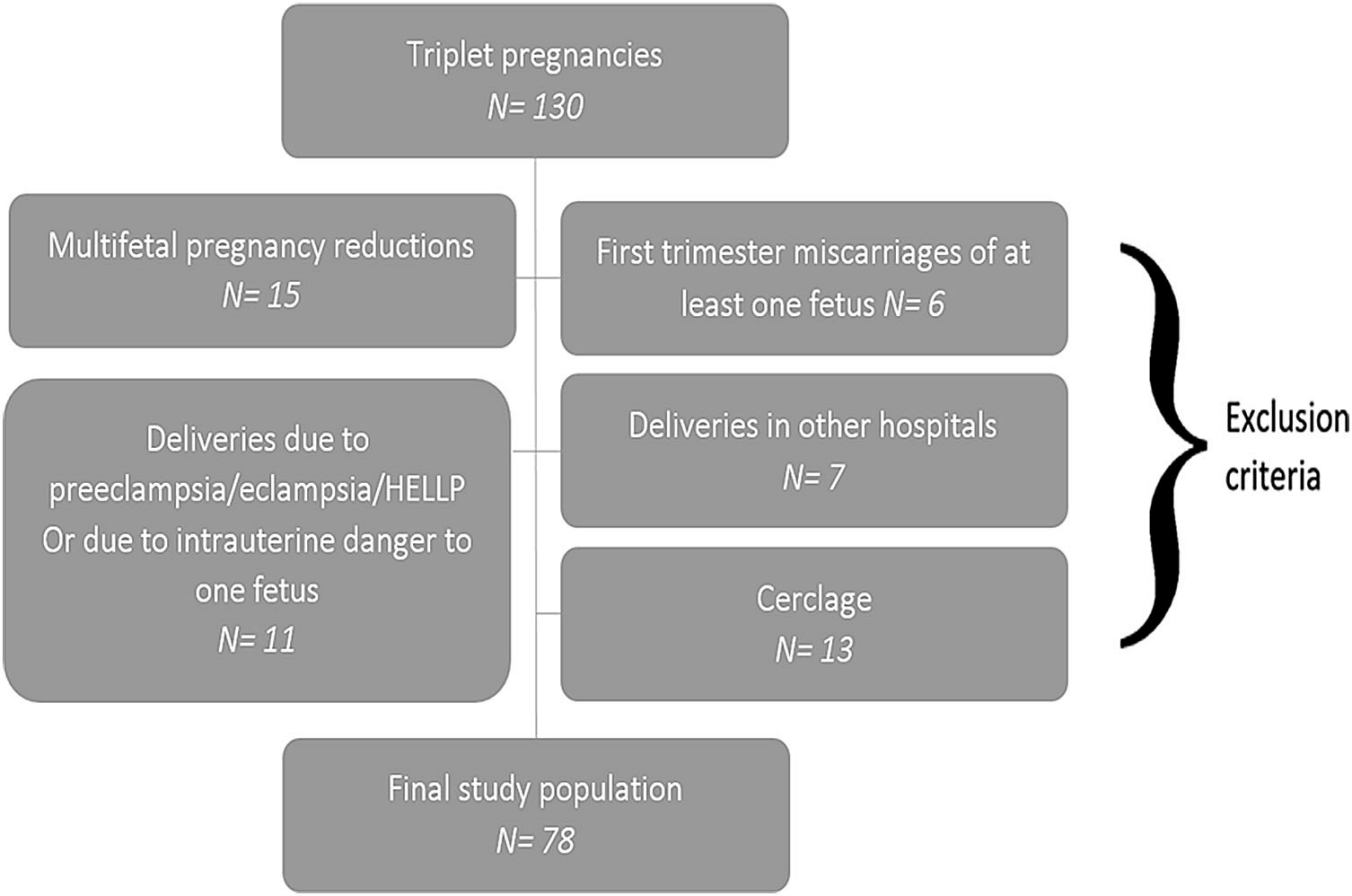
Study flow chart

In this retrospective analysis, we studied 130 women who were diagnosed with a triplet pregnancy at the time of the first trimester screening from January 2003 to August 2015 (*n* = 130). The following patients were then excluded from the study: Seven women did not deliver at the department and had no follow-up (*n* = 7). Eleven cases with PTD before 32 + 0 weeks of gestation due to other reasons, namely preeclampsia/eclampsia, the HELLP syndrome (hemolysis, elevated liver enzymes, low platelet count), and an imminent intrauterine danger to one of the fetuses’ life (*n* = 11) were also excluded. Thirteen women had undergone cerclage and were, thus, also excluded from the analysis. Moreover, we excluded all women who underwent multifetal pregnancy reduction (*n* = 15) or had a first trimester miscarriage of at least one fetus (*n* = 6). This means that only cases that started with triplets and gave birth to triplets were included. The study examines spontaneous preterm deliveries as opposed to iatrogenic ones. This resulted in a final study population of 78 triplet pregnancies for this analysis of serial CL measurements. A flow chart is depicted in Fig. 2.

Relevant data were acquired retrospectively using the Viewpoint^®^ software (GE Healthcare, Wessling, Germany). This is the basic perinatologic database used at the department. CL measurements were performed every 2 weeks from 16 + 0 until 30 + 0. In addition, the following parameters were included: history of previous conization procedures, age at delivery, body mass index (BMI) at the initial visit, parity, previous preterm birth due to cervical insufficiency, preterm labor, or preterm premature rupture of membranes, previous second trimester miscarriage, pregnancies after in vitro fertilization (IVF), cigarette smoking, and chorionicity (pregnancies were divided into mono-/dichorionic and trichorionic).

This study was approved by the Institutional Review Board of the Medical University of Vienna (IRB Number: 1602/2015) on April 14th 2016. Neither written nor verbal informed consent is necessary in retrospective studies according to the Ethics Committee of the Medical University of Vienna and was thus not obtained. The data were de-identified for statistical analysis.

Nominal variables are reported as numbers and frequencies, and continuous variables as medians and interquartile ranges (IQR). Paired *t* tests were applied to test for differences between subsequent CL measurements within one group. An unpaired *t* test was used to test for the differences in initial CL between women with and without early PTD. For *t* tests, data had to be normally distributed as evaluated by the Kolmogorov–Smirnov test. This was the case for all of these analyses. For *t* tests, the *t* value and the degree of freedom (*df*) are provided. In a stepwise linear regression model for prediction of PTD <32 + 0, we included the following parameters: basic patient characteristics, pregnancy-related parameters including chorionicity and previous conization procedures, and CL measurements as chosen according to the analyses of CL dynamics and to clinical considerations (see below). These analyses were performed to allow an early prediction of PTD <32 + 0. The optimal cut-off for CL was calculated as the threshold value with the highest specificity and sensitivity based on the receiver-operating characteristics (ROC) curve as a sensitivity versus (1 − specificity) plot. This is associated with the highest discriminatory ability. The discriminatory ability of the investigated parameters is described as the correlation between specificity and sensitivity, and was measured by the area under the receiver-operating curve (AUC). Where appropriate, values are given with a 95% confidence interval (95% CI). Statistical analysis was performed using SPSS Statistics for Windows, Version 17.0 (SPSS Inc., Chicago, USA). Differences were considered statistically significant if *p* < 0.05.

## Results

The median gestational age at delivery was 32 completed weeks (IQR 29–33). 16 (20.5%) women, delivered between 23 + 0 and 32 + 0 weeks of gestation. Four women (5.1%) suffered a late miscarriage (17 + 0 to 18 + 6 weeks) and 6 women (7.7%) delivered a premature infant with lifesigns between weeks 19 + 0 and 23 + 0. This resulted in an overall rate of 33.3% (26/78) for early PTD <32 + 0 weeks used for the following analyses. Details on patient characteristics are shown in Table 1.

**Table 1:** Basic patient characteristics

Age (years)^a^	31 (27; 35)
Body mass index (kg/m^2^)^a^	22.8 (21.0; 25.7)
Previous second trimester miscarriage or preterm delivery^b^	7 (9.0)
Previous conization^b^	3 (3.8)
Pregnancy after IVF treatment^b^	53 (67.9%)
Parity^b^	
0	55 (70.5)
1	18 (23.1)
≥2	5 (6.4)
Cigarette smoking during pregnancy^b^	9 (11.5)
Chorionicity^b^	
1	1 (1.3)
2	22 (28.2)
3	55 (70.5)
Amniocity^b^	
1	0
2	0
3	78 (100)

Data are presented as ^a^ median (interquartile range) or ^b^ numbers (frequencies)

**Fig. 3:**
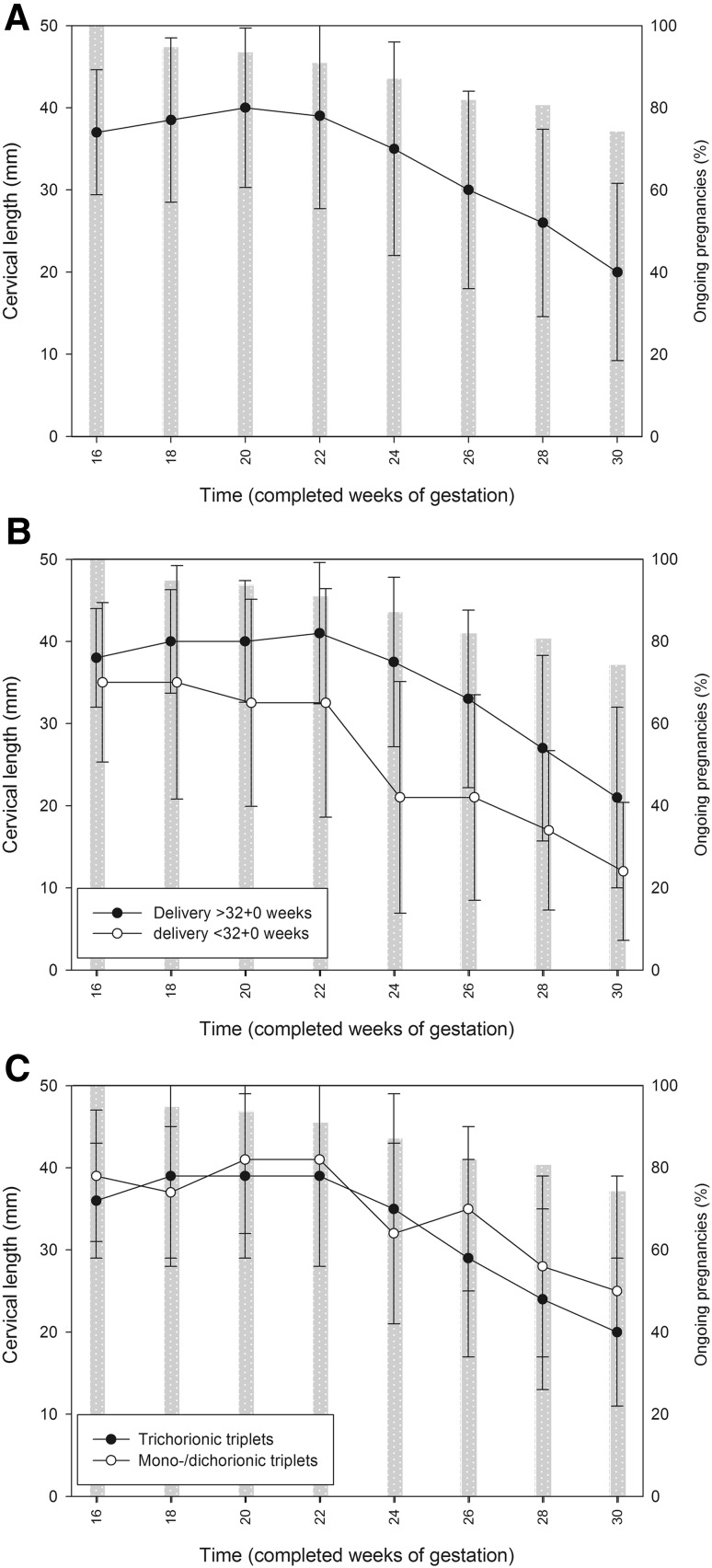
Dynamics in cervical lengths in the course of routine screening in triplet pregnancies (data provided as median and standard deviation). **a** Complete study population. **b** Women with (*n* = 37, *white dots*) and without preterm delivery <32 + 0 weeks of gestation (*n* = 54, *black dots*). The group of women with preterm delivery includes those with a second trimester miscarriage. **c** Women with mono-/dichorionic triplets (*n* = 23, *white dots*) and trichorionic triplets (*n* = 55, *black dots*). The *gray bars* indicate how many pregnancies were ongoing at this time of measurement (provided in %). Gestational age is plotted on the *x*-axis

Dynamics in CL are plotted in Fig. 3. Due to the second trimester miscarriages and the preterm deliveries, only those with an ongoing pregnancy were available for CL measurement at follow-up examinations. In detail, in week 16 + 0, all women underwent CL measurements, 74 (94.9%) in week 18 + 0, 73 (93.6%) in week 20 + 0, 71 (91.0%) in week 22 + 0, 68 (87.2%) in week 24 + 0, 64 (82.1%) in week 26 + 0, and 63 (80.8%) in week 28 + 0. For the whole study population, a stable CL course was seen from week 16 + 0 (median 37 mm, IQR 34–40) to week 22 + 0 (median 39 mm, IQR 33–43; *p* = 0.773, *t* = 0.291, *df* = 39). Thereafter, significant declines in CL were noted with median differences of 4–5 mm every 2 weeks: median CL was 35 mm (IQR 26–42; *p* < 0.001, *t* = 4.090; *df* = 42) in week 24 + 0 and declined to 30 mm (IQR 20–36; *p* < 0.001, *t* = 3.943; *df* = 38) in week 26 + 0; in week 28 + 0, it had further decreased to a median 26 mm (IQR 16–32; *p* < 0.001, *t* = 4.868; *df* = 45) and to a median of 20 mm (IQR 14–26; *p* = 0.009, *t* = 2.752; *df* = 40) in week 30 + 0 (Fig. 3a).

The comparison of CL measurements between women with (*n* = 26) and without PTD <32 + 0 weeks (*n* = 52) is shown in Fig. 3b. In week 16 + 0, groups did not differ in CL (35 mm, IQR 28–39, vs. 38 mm, IQR 35–41, respectively; *p* = 0.058, *t* = −1.935, *df* = 53). When focusing on women with PTD <32 + 0 weeks, a significant difference in CL was found only between weeks 22 + 0 and 24 + 0 (median 33 mm, IQR 17–39 vs. 21 mm, IQR 7–30; *p* = 0.005, *t* = 3.440, *df* = 12). Women without PTD <32 + 0, showed significant 2-week differences from week 22 + 0 on (*p* < 0.05).

A logistic regression model was computed to analyze predictive factors for PTD <32 + 0 (Table 2). In addition to the general patient and pregnancy characteristics, we chose to include CL at weeks 16 + 0, 18 + 0, 20 + 0, 22 + 0, and 24 + 0 as well as CL dynamics between weeks 22 + 0 and 24 + 0, since the latter revealed a significant CL decline also for women with PTD <32 + 0 (see above). The logistic regression models’ univariate analyses revealed that all CL parameters differed significantly between women with and without PTD <32 + 0 except CL in weeks 16 + 0 and 18 + 0. In multivariate analyses, all parameters that had been significant in the univariate models were included except CL dynamics between weeks 22 + 0 and 24 + 0 due to redundancy. However, none reached statistical significance (Table 2).

**Table 2:** Prediction of early preterm delivery in women with a triplet pregnancy

	Delivery <32 + 0 week (*n* = 37)	Delivery ≥32 + 0 week (*n* = 54)	Univariate analysis			Multivariate analysis		
			OR (95% CI)^a^	Wald’s test	*p* (LR test)^b^	Adjusted OR (95% CI)^a^	Wald’s test	*p* (LR test)^b^
Age (years)^c^	31.5 (26.8; 37.0)	31.0 (27.0; 33.0)	1.04 (0.95; 1.13)	0.644	0.422	–	–	–
Body mass index (kg/m^2^)^c^	23.9 (20.6; 26.7)	22.5 (21.0; 25.4)	1.03 (0.91; 1.17)	0.278	0.598	–	–	–
Chorionicity (3 versus 1 or 2)^d^	21 (80.8)	35 (67.3)	2.04 (0.66; 6.35)	1.517	0.218			
Previous preterm delivery or second trimester miscarriage^d^	4 (15.4)	3 (5.8)	2.97 (0.61; 14.41)	1.825	0.177	–	–	–
Previous conization^d^	1 (3.8)	2 (3.8)	1.00 (0.09; 11.57)	0.000	1.000	–	–	–
Pregnancy after IVF treatment^d^	16 (61.5)	37 (71.2)	0.65 (0.24; 1.75)	0.731	0.392	–	–	–
Parity^d^								
0	18 (69.2)	37 (71.2)	Reference	1.706		–	–	–
1	5 (19.2)	13 (25.0)	0.79 (0.24; 2.56)	0.154	0.426	–	–	–
≥2	3 (11.5)	2 (3.8)	3.08 (0.47; 20.1)	1.384		–	–	–
Cigarette smoking^d^	3 (11.5)	6 (11.5)	1.00 (0.87; 1.15)	0.000	1.000	–	–	–
CL^e^ at week 16 + 0 (mm)^c^	35 (28; 39)	38 (35; 41)	0.93 (0.85; 1.01)	3.146	0.058	–	–	–
CL^e^ at week 18 + 0 (mm)^c^	35 (29; 41)	40 (35; 44)	0.93 (0.87; 1.01)	3.381	0.066	–	–	–
CL^e^ at week 20 + 0 (mm)^c^	33 (28; 42)	40 (36; 45)	0.90 (0.82; 0.98)	6.276	*0.012* ^f^	1.04 (0.84; 1.29)	0.131	0.717
CL^e^ at week 22 + 0 (mm)^c^	33 (17; 39)	41 (36; 43)	0.92 (0.68; 0.98)	6.958	*0.008* ^f^	1.01 (0.76; 1.36)	0.006	0.937
CL^e^ at week 24 + 0 (mm)^c^	21 (7; 30)	38 (31; 42)	0.91 (0.86; 0.97)	9.873	*0.002* ^f^	0.84 (0.68; 1.03)	7.262	0.097
CL^e^ dynamics: week 22 + 0 to 24 + 0 (mm)^c^	−10.5 (−16.0; −1.6)	−2.5 (−7.0; 1.0)	0.88 (0.79; 0.97)	6.043	*0.014* ^f^	*Excluded for redundancy*		

Results of the univariate and multivariate analysis

^a^OR (95% CI) = odds ratio (95% confidence interval)

^b^LR test = likelihood ratio test

^c^Continuous variable, provided in median (interquartile range)

^d^Nominal variable, provided in *n* (%)

^e^CL = cervical length

^f^Italic letters indicate statistical significance

**Fig. 4:**
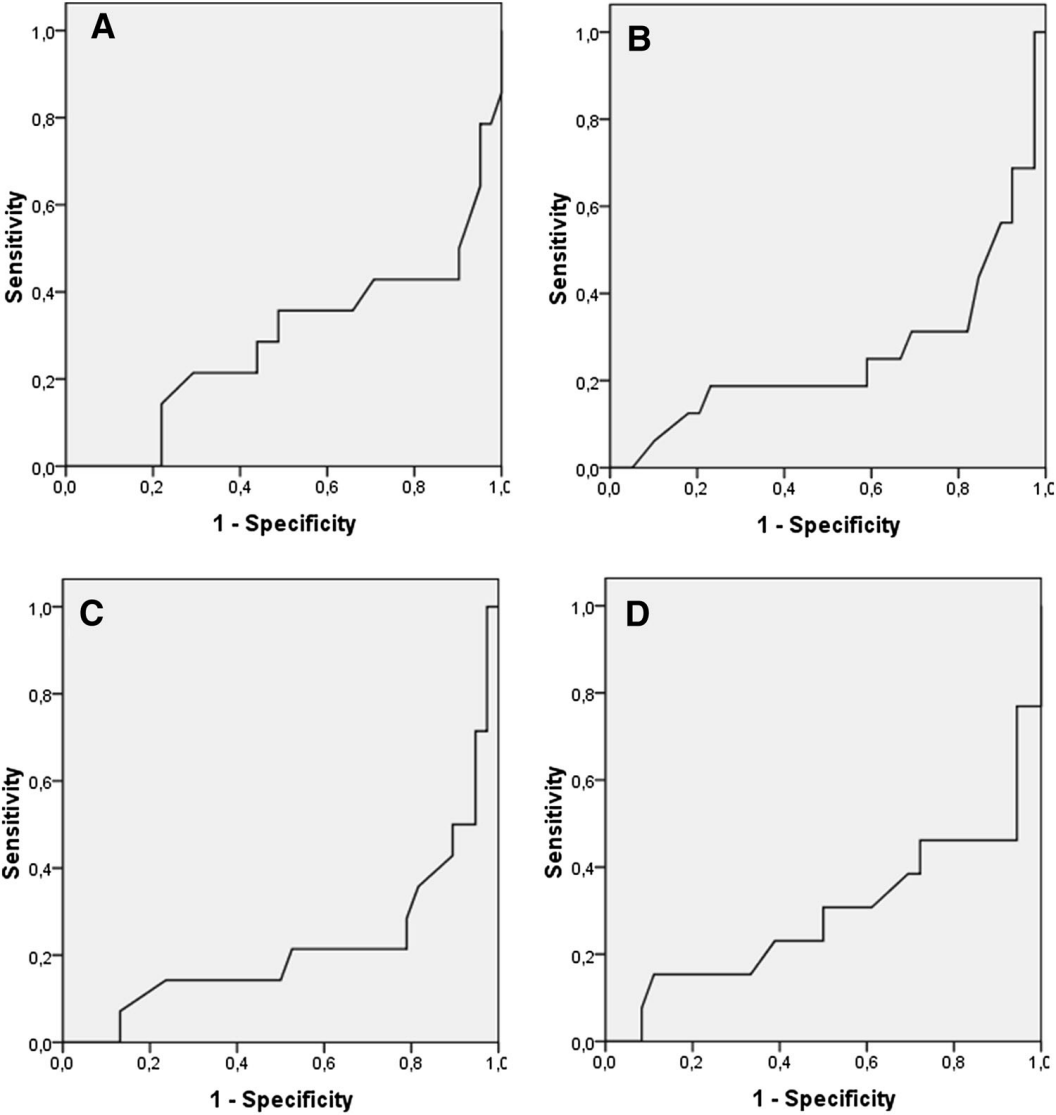
Receiver-operating characteristic *curves* for the prediction of preterm delivery <32 + 0 weeks using the following cervical length cut-off values: **a** <34 mm for week 20 + 0; **b** < 34 mm for week 22 + 0; **c** <35 mm for week 24 + 0; and **d** a decrease between weeks 22 + 0 and 24 + 0 exceeding 10 mm

For all significant predictors (Table 2), the following optimized cut-off values for the prediction of PTD <32 + 0 were calculated (see Fig. 4 for receiver-operating characteristic curves): <34 mm for week 20 + 0 (sensitivity 0.57, 95% CI 0.29–0.82; specificity 0.90, 95% CI 0.77–0.97; positive predictive value 0.67, 95% CI 0.35–0.90; negative predictive value 0.86, 95% CI 0.72–0.95), <34 mm for week 22 + 0 (sensitivity 0.69, 95% CI 0.41–0.89; specificity 0.82, 95% CI 0.66–0.92; positive predictive value 0.61, 95% CI 0.36–0.83; negative predictive value 0.86, 95% CI 0.71–0.95), <35 mm for week 24 + 0 (sensitivity 0.79, 95% CI 0.49–0.95; specificity 0.79, 95% CI 0.63–0.90; positive predictive value 0.58, 95% CI 0.34–0.80; negative predictive value 0.91, 95% CI 0.76–0.98), and a decrease between weeks 22 + 0 and 24 + 0 exceeding 10 mm (sensitivity 0.54, 95% CI 0.25–0.81; specificity 0.94, 95% CI 0.81–0.99; positive predictive value 0.78, 95% CI 0.40–0.97; negative predictive value 0.85, 95% CI 0.70–0.94).

In addition, the influence of chorionicity on CL was evaluated. The according graphs are plotted in Fig. 3c. No differences in CL between trichorionic and mono-/dichorionic triplets were found at any gestational age (*p* > 0.05). In mono-/dichorionic pregnancies (*n* = 23), significant declines in CL were found between weeks 26 + 0 and 28 + 0 (35 mm, IQR 22–38, vs. 28 mm, IQR 16–32; *p* = 0.024, *t* = 2.528, *df* = 14) and 28 + 0 and 30 + 0 (28 mm, IQR 16–32, vs. 25 mm, IQR 10–33; *p* = 0.042, *t* = 2.261, *df* = 13). In trichorionic pregnancies (*n* = 55), CL shortened in a statistically significant manner between weeks 22 + 0 and 24 + 0 (39 mm, IQR 33–42, vs. 35 mm, IQR 25–41; *p* = 0.001, *t* = 3.784, *df* = 30), 24 + 0 and 26 + 0 (35 mm, IQR 25–41, vs. 29 mm, IQR 19–36; *p* = 0.001, *t* = 3.590, *df* = 27), and 26 + 0 and 28 + 0 (29 mm, IQR 19–36, vs. 24 mm, IQR 16–32; *p* < 0.001, *t* = 4.111, *df* = 30).

## Comment

This retrospective analysis on CL in triplet pregnancies provided three major results: (1) from week 22 + 0 onwards, a decrease of CL in the overall study population could be observed; (2) triplet pregnancies resulting in second trimester miscarriages and PTD <32 + 0 weeks showed a pronounced decrease in CL between weeks 22 + 0 and 24 + 0; and (3) significant predictors of PTD <32 + 0 are the CL in the 20th, 22nd, and 24th week of gestation, as well as the dynamics of CL from the 22 + 0 to the 24 + 0 week.

In our dataset, triplet pregnancies resulted in PTD from weeks 23 + 0 to 31 + 6 in 20.5% and in second trimester miscarriage in 12% with all of these cases due to cervical, preterm labor, or preterm premature rupture of membranes only. This is comparable to the literature reporting rates of 13–64.7% [4, 15] with the majority of incidences ranging from 16 to 31.4% [10, 11, 13, 14].

As demonstrated in Fig. 3, a certain decline of CL appears to be physiological in triplet pregnancies. From week 22 + 0 on, significant 2-week declines in CL were found for the whole study population as well as for women without PTD <32 + 0. This finding is in accordance with previous studies [15, 19]. Ramin et al. also reported that progressive cervical shortening was visible only after week 20 + 0 [15]. Hiersch et al. observed a more rapid cervical shortening during gestation in triplets compared to twin pregnancies [18]. However, a sharp decline in cervical length from week 22 + 0 to 24 + 0 seems to be specific for women with ongoing triplet pregnancies at risk for PTD between 24 + 0 and 32 + 0 weeks (Table 2). The predictive value of CL on PTD has already been demonstrated in previous studies [2, 4, 8, 9, 11–16, 19]. Notably, optimized cut-off values for absolute CL were 34–35 mm in our study which resulted in sensitivity and specificity values of 60–80 and 70–90%, respectively. Previous studies on triplet pregnancies often used a lower cut-off value of 25 mm [14, 20] or calculated similar optimized cut-off values [11–13]. We find it hard to comment on these differences. In some studies, the lower cut-off level is likely related to the fact that standardized measurement was performed in a higher week of gestation and, thus, related to the physiological decline in CL [15, 19]. Notably, the optimized cut-off values in our study provide quite high negative predictive values (nearly 90% in weeks 20 + 0, 22 + 0, and 24 + 0) which are reassuring for the patient. Only van den Mheen et al. used a higher CL cut-off of 30 mm for the prediction of PTD <32 + 0 weeks [4]. Applying this criterion, they achieved a similar negative predictive value of 87.4%. It seems reasonable that a negative predictive value of 100% would be desirable since it would provide maximum stress relief for the pregnant woman. In a previous study, this could be reached with a cut-off level of 20 mm in weeks 25–28 [14]. Unfortunately, our results do not prove these findings.

None of the CL parameters mentioned above remained significant predictors in the multivariate model (Table 2). It seems reasonable to assume a certain redundancy of values. Moreover, the analysis is influenced by the fact that women with a shorter CL might have already experienced second trimester miscarriage or PTD and could therefore not be included in the subsequent CL measurements. However, each of these CL parameters seems to have its relevance in clinical routine and none of them should be considered more accurate and meaningful than the others.

Concerning the analysis of CL dynamics in women with mono-/dichorionic versus trichorionic triplets (Fig. 3c), no striking differences between these two groups were found. The 2-week CL decreases from week 22 + 0 on reached statistical significance in the trichorionic group more often which is likely due to the smaller patient population with mono-/dichorionic triplets. Moreover, chorionicity was not a predictive parameter for PTD <32 + 0 in the logistic regression model (Table 2) which somehow is in contrast to a recent report [7]. Thus, our data suggest that chorionicity is of only minor impact on CL. However, larger studies need to clarify this topic.

The main strength of our study is the large sample size. To the best of our knowledge, this is the largest study on CL in triplet pregnancies so far. Moreover, CL screening was rigorous and evaluation of CL dynamics with 2-week intervals might have improved data validity. The limitations of the study are its retrospective design and the lack of fetal fibronectin testing. Since this method is now being routinely applied, more complete datasets may be available in the future. This is due to the historical population and the fact that some of these procedures had been performed outside of the center.

In conclusion, in triplet pregnancies, a decrease in CL seems physiological from week 22 + 0 onwards. A sharp decline in CL from the 22 + 0 to the 24 + 0 week increases the risk of PTD <32 + 0 weeks. Optimized cut-off values for CL in weeks 20 + 0, 22 + 0, and 24 + 0 were 34–35 mm. Further study on CL dynamics in triplet pregnancies is recommended in order to develop multivariate predictive models with higher reliability.
